# Conceptual and theoretical dimensions of biodiversity research in China: examples from plants

**DOI:** 10.1093/nsr/nwab060

**Published:** 2021-04-15

**Authors:** Chengjin Chu, Lei Chen, Pengfei Fan, Ziwen He, Yuanzhi Li, Jinbao Liao, Xubing Liu, Kechang Niu, Xingfeng Si, Shaopeng Wang, Xinqiang Xi

**Affiliations:** State Key Laboratory of Biocontrol and School of Life Sciences, Sun Yat-sen University, China; State Key Laboratory of Vegetation and Environmental Change, Institute of Botany, Chinese Academy of Sciences, China; State Key Laboratory of Biocontrol and School of Life Sciences, Sun Yat-sen University, China; State Key Laboratory of Biocontrol and School of Life Sciences, Sun Yat-sen University, China; State Key Laboratory of Biocontrol and School of Life Sciences, Sun Yat-sen University, China; Key Laboratory of Poyang Lake Wetland and Watershed Research, Jiangxi Normal University, China; State Key Laboratory of Biocontrol and School of Life Sciences, Sun Yat-sen University, China; Department of Ecology, School of Life Sciences, Nanjing University, China; Zhejiang Tiantong Forest Ecosystem National Observation and Research Station, School of Ecological and Environmental Sciences, East China Normal University, China; Institute of Ecology, College of Urban and Environmental Sciences, and Key Laboratory for Earth Surface Processes of the Ministry of Education, Peking University, China; Department of Ecology, School of Life Sciences, Nanjing University, China

The search for the underlying mechanisms of species origin, maintenance and extinction is among the highest priorities in biodiversity science. In parallel with the rapid accumulation of empirical evidence for existing theories in this field in China [1], numerous conceptual and theoretical achievements of basic biodiver-sity science have been made, accompanied by methodological breakthroughs. These advances have greatly contributed to evolution of the theoretical frameworks of ecology and conservation biology and guided biodiversity management, and, ultimately, could help achieve the vision of ecosystem health and sustainability. Here we focus on the basic biodiversity research in China through the lens of plants. Even with this limited scope, the studies listed here are not exhaustive but an attempt to partly demonstrate the current status of basic biodiversity research in China. Accordingly, this *perspective* is organized into two closely relevant sections, that is biodiversity origination and maintenance, and concludes with future research directions.

Speciation is the starting point of biodiversity. It has been depicted in multiple modes, such as allopatric, sympatric and parapatric/peripatric speciation. Exploring speciation processes in biodiversity hotspots helps us reveal the formation and evolutionary mechanisms of species diversity. Based on extensive investigations of mangrove species in the Indo-western Pacific area, He *et al.* [2] proposed and verified a novel speciation mechanism: the mixing-isolation-mixing cycles model, which can promote speciation in more geographical features compared to classical models. It provides a new viewpoint to explain the formation of global biodiversity hotspots. In addition, hybrid speciation is also an important source of new species. Researchers proposed a universal molecular genetic model for homoploid hybrid speciation, which is critical for understanding the formation of hybrid species (Supporting information (SI), ref. 1).

In the past two decades in China, research has focused on the maintenance of biodiversity, especially species diversity, rather than formation. Methodologically, several sampling approaches have been developed with the aim of more accurately estimating population sizes and species distribution, and tracking seed dispersal (SI, refs. 2–4), which offer new opportunities for basic biodiversity research. Furthermore, we identified four key ongoing shifts in the field greatly contributed by Chinese researchers: (i) from direct to indirect biotic interactions, (ii) from interspecific to intraspecific trait variations, (iii) from single to multiple trophic levels, and (iv) across multiple spatial scales rather than at a single scale.

Biotic interactions, both direct and indirect, are fundamental to population dynamics, the maintenance of biodiversity and ecosystem functioning. Numerical and experimental manipulations developed recently have contributed substantially to understanding the role of direct interactions. Using species interactions networks constructed from the BEF-China experiment, a method termed ‘directed species loss’ was introduced to separate the effects of node loss and link loss of networks on ecosystem functioning, and it was found that directed species loss could severely hamper productivity in already diverse young plantation forests (SI, ref. 5). Using mesh-walled in-growth cores possessing different size of nylon mesh to implement the hyphal exclusion, a recent study tested the specific role of common mycorrhizal networks on demography of ectomycorrhizal and arbuscular mycorrhizal tree seedlings (SI, ref. 6). Additionally, by combining long-term seedling census dataset and high throughput sequencing, Chen *et al.* [3] investigated the role of species mycorrhizal types and soil fungi in mediating tree neighborhood interactions. These methods will ultimately facilitate unravelling of the effects of different functional types of soil microbes on species coexistence over various spatiotemporal scales, especially in highly diverse forests.

Species coexistence, the most essential aspect of biodiversity science, has focused overwhelmingly on mechanisms operating between species pairs. However, such frameworks do not always perform well in characterizing population dynamics, partially as a result of the ignorance of complex indirect interactions. Interactions are indirect if the effect of one species on another depends on changes in density or trait of other species, and can be further classified as ‘interaction chains’ (ICs, also termed ‘density-mediated indirect interactions’) and ‘higher-order interactions’ (HOIs, also termed ‘trait-mediated indirect interactions’). To this end, Li *et al.* [4] constructed a metapopulation model incorporating both competitive intransitivity (a type of IC, like the game ‘rock-paper-scissors’) and HOIs. This was the first theoretical study to explore simultaneously the effects of these two types of indirect interactions on biodiversity maintenance. They found that the ICs increased biodiversity by preventing the dominance of some species, while the HOIs facilitated species coexistence through stabilizing community fluctuations. By developing a novel approach to quantify the individual-level HOIs with the inclusion of size and distance among individuals, Li *et al.* [5] found that the various existing definitions of HOIs can actually be reconciled at the individual level, and provided the first empirical evidence of HOIs determining the survival and growth of trees in a temperate forest. In addition, a patch-dynamic model (SI, ref. 7) was developed for the plant-fly-wasp tripartite network observed in the eastern Tibetan Plateau (SI, ref. 8), which is the first attempt to characterize the dynamics of whole tripartite metacommunities interacting in realistic higher-order ways (Fig. 1).

Despite ecological differences among species being proposed as the fundamental condition for biodiversity maintenance, the neutral theory of biodiversity that emerged at the outset of this century challenged such classic paradigm by successfully predicting many biodiversity patterns under the assumption of functional equivalence among individuals. A theoretical study showed that two seemingly identical species with local mate competition and population size-dependent sex ratio adjustment may coexist, which was further exemplified by the cryptic species of fig wasps (SI, ref. 9). By proposing a nearly neutral model characterized by birth-death tradeoffs among species, Zhou and Zhang [6] made a key step forward to reconcile well-observed interspecific differences with the merit of neutral theory, which substantially alleviated the criticism from experimental researchers on the theory, and uniquely advanced the field of tradeoffs-based community ecology. By integrating species traits and phylogenetic relationships, researchers extended the classic Theory of Island Biogeography to community structure measures (SI, ref. 10), providing a new way to account for nonrandom processes beyond the assumption of species equivalence.

**Figure 1.**
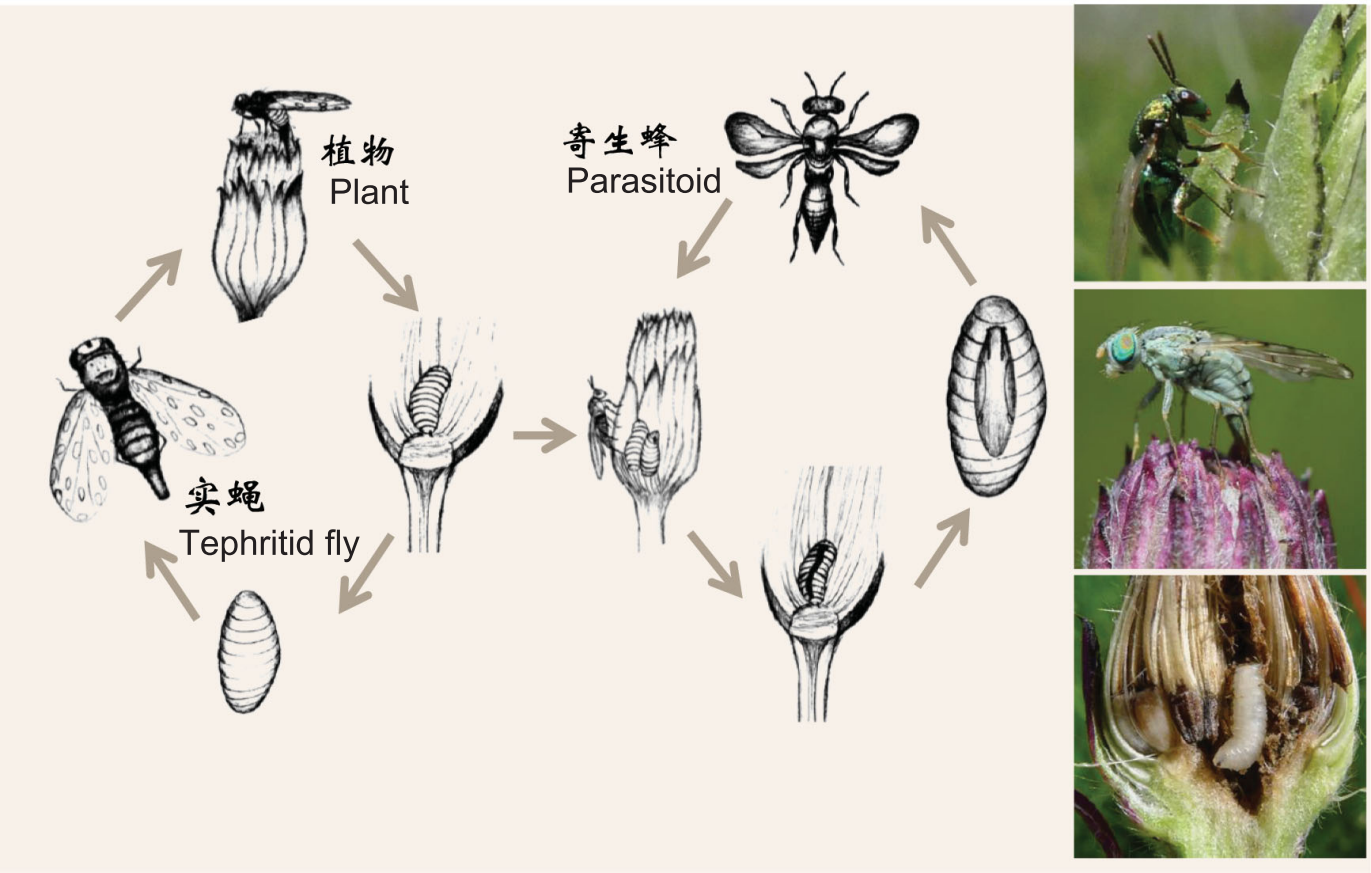
‘Plants-Tephritid fly seed predators-Parasitoids’ co-evolved tri-trophic level system in the eastern Tibetan Plateau. A recent study revealed that the host use of parasitoids was determined not only by traits of herbivores, but also, to a large extent, by the plant flowerhead diameter (SI, ref. 8). Consequently, larvae of the same tephritid fly species in different plant species are actually different host resources for parasitoid communities. Such divergence in plant species may create new niches for herbivores, and also result in multiplicative increase in parasitoids in non-neighboring trophic levels, indicating the importance of species diversity itself acting as the ‘seeds of diversity’. Researchers theoretically demonstrated that plant-mediated resource partitioning among parasitoids resulted in the highest species diversity under intermediate levels of disturbance in this tri-trophic level system, offering insights into how realistic higher-order interactions structure the complex multipartite networks (SI, ref. 7). Photo credit: Xinqiang Xi.

Besides the well-recognized interspecific trait differences, there is increasing interest in the functional significance of intraspecific trait variability (ITV), which results from genetic adaption and phenotypic plasticity, in organizing individual performance, community assembly and ecosystem functioning. Yet, it remains a challenge to quantify the interaction and relative importance of interspecific versus intraspecific trait variation in natural communities. Drawing on a novel conceptual framework, Niu *et al.* [7] predicted that importance of ITV increased with environmental harshness because of increasing phenotypic and genotypic diversity. Using a novel analytic method, they distinguished functional trait diversity attributable to ITV versus interspecific trait variation. They further illustrated that the ITV promoted by the eco-evolutionary processes can partly offset the loss of interspecific trait diversity, which can act as a buffering mechanism stabilizing the functional structure of communities along environmental gradients. Nevertheless, the functional significance of genetic variability *per se* in community assembly remains unclear. By genotyping all individuals of a tropical tree species, *Beilschmiedia roxburghiana*, in a 20-ha tropical seasonal rainforest dynamics plot in Xishuangbanna, China, researchers showed that genetically related individuals were often spatially clustered but neighbored by close relatives, which reduced greatly the growth performance of focal trees, that is there was a stronger conspecific negative density dependence (CNDD) possibly promoting species coexistence via rare genotype advantage (SI, ref. 11). Furthermore, it was found recently that genetic richness affected trait variation but not community productivity, in the BEF-China tree diversity experiment conducted at the Xingang Mt., China (SI, ref. 12). These two studies set an excellent background on how to link genetic variability with key concepts such as CNDD in ecology, and shed light on developing an integrative framework from genes to eco- systems.

Organisms live in a complex network connected by interactions both within and across trophic levels. Food webs characterize the trophic interactions between species within an ecosystem. The structure of food webs has long been recognized to influence the maintenance of biodiversity and ecosystem functioning. However, previous studies have primarily explored small trophic modules, for example food chains, in contrast to the complexity of natural ecosystems. Several studies have developed complex food web models and obtained novel theoretical insights. Wang and Brose [8] demonstrated that ecosystem primary productivity increased exponentially with the maximum trophic level, highlighting the role of top predators in regulating ecosystem functioning. Such a ‘vertical diversity hypothesis’ contributes to extending the biodiversity-ecosystem functioning theory to multitrophic systems. As a result of global change and anthropogenic disturbances, exploring the separate effects of habitat loss and fragmentation (two main processes of habitat destruction) on spatial food web persistence has become a fundamental issue in ecology and conservation. Using a pair approximation approach that distinguished habitat fragmentation from habitat loss, Liao *et al.* [9] constructed a spatial patch-dynamic framework for food webs by incorporating trophic-dependent dispersal, and found that species display diverse responses (negative, neutral or positive) to habitat loss and fragmentation separately.

Scale dependence penetrates almost every ecological pattern and process. Extrapolating or interpolating results obtained at a given scale to others can be extremely useful but rather challenging. Whether biodiversity begets stability is a long-standing debate, which has stimulated much interest among both theoretical and experimental researchers. However, studies so far have mostly focused on biodiversity and stability in local ecosystems, and whether or not predictions from these local-scale studies can extend to larger spatial scales remains unclear. To address this issue, Wang *et al.* [10] constructed consistent metrics of temporal stability from local to regional scales and predicted that local biodiversity (alpha diversity) and species turnover over space (beta diversity) both provided insurance effects for ecosystem stability across scales. Such a framework provides a novel tool for understanding the scale dependency of diversity–stability relationships and has been applied to a wide range of study systems (SI, refs. 13–14).

As summarized above, although not exhaustively, in the last two decades especially the past five years, we have seen a rapid development of basic biodiversity research in China, with some novel conceptual and theoretical achievements along with new methodological development. With the unprecedented boost of basic research regarding ecological civilization, and the rejuvenated recognition of the fact that everything is interconnected and interdependent in nature (i.e. the entangled bank [SI, ref. 15] simplified in Fig. 1), we believe that there will be more breakthroughs in basic biodiversity science. Specifically, we suggest that future research should focus on the following three aspects: (i) developing new theory to advance our understanding of the relationships between structure, diversity and functioning of ecosystems across spatiotemporal scales and trophic levels in the context of ecological networks; (ii) exploring the effects of biotic interactions (direct and indirect) and abiotic factors on co-evolution among organisms (as exemplified in Fig. 1), and the impact on biodiversity; (iii) developing a complete spectrum from genes to ecosystems, to test the community- and ecosystem-level consequences of speciation and genetic diversity, and vice versa, which will further integrate studies on species origination and maintenance via eco-evolutionary models.

## Supplementary Material

nwab060_Supplemental_File
