# Supplementary material for: Conceptual and theoretical dimensions of biodiversity research in China: examples from plants
Source: Natl Sci Rev. 2021 Apr 15;8(7):nwab060. doi: 10.1093/nsr/nwab060 (PMC8310762; doi:10.1093/nsr/nwab060)
Supplement: nwab060_Supplemental_File [file nwab060_supplemental_file.docx]

**SUPPORTING INFORMATION**

**REFERENCES**

1. Wang Z, Jiang Y, Bi H et al. Hybrid speciation via inheritance of alternate alleles of parental isolating genes. *Molecular Plant*. 2021; **14**: 208-222.

2. Xiao Z, Jansen PA and Zhang Z. Using seed-tagging methods for assessing post-dispersal seed fate in rodent-dispersed trees. *Forest Ecology and Managemen*t. 2006; **223**: 18-23.

3. He F and Hubbell SP. Species–area relationships always overestimate extinction rates from habitat loss. *Nature*. 2011; **473**: 368-71.

4. Qiao H, Peterson AT, Ji L et al. Using data from related species to overcome spatial sampling bias and associated limitations in ecological niche modelling. *Methods in Ecology and Evolution*. 2017; **8**: 1804-12.

5. Chen Y, Huang Y, Niklaus PA et al. Directed species loss reduces community productivity in a subtropical forest biodiversity experiment. *Nature Ecology & Evolution*. 2020; **4**: 550-9.

6. Liang M, Johnson D, Burslem DFRP et al. Soil fungal networks maintain local dominance of ectomycorrhizal trees. *Nature Communications*. 2020; **11**: 2636.

7. Liao J, Xi X, Bearup D et al. Metacommunity robustness of plant–fly–wasp tripartite networks with specialization to habitat loss. *Ecology*. 2020; **101**: e03071.

8. Xi X, Yang Y, Yang Y et al. Plant-mediated resource partitioning by coexisting parasitoids. *Ecology*. 2017; **98**: 1660-70.

9. Zhang D, Lin K and Hanski I. Coexistence of cryptic species. *Ecology Letters*. 2004; **7**: 165-9.

10. Si X, Cadotte MW, Zeng D et al. Functional and phylogenetic structure of island bird communities. *Journal of Animal Ecology*. 2017; **86**: 532-42.

11. Shao X, Brown C, Worthy SJ et al. Intra-specific relatedness, spatial clustering and reduced demographic performance in tropical rainforest trees. *Ecology Letters*. 2018; **21**: 1174-81.

12. Bongers FJ, Schmid B, Durka W et al. Genetic richness affects trait variation but not community productivity in a tree diversity experiment. *New Phytologist*. 2020; **227**: 744-56.

13. Mehrabi Z and Ramankutty N. Synchronized failure of global crop production. *Nature Ecology & Evolution*. 2019; **3**: 780-6.

14 Lamy T, Wang S, Renard D et al. Species insurance trumps spatial insurance in stabilizing biomass of a marine macroalgal metacommunity. *Ecology*. 2019; **100**: e02719.

15. Darwin C. *On the Origin of Species by Means of Natural Selection*. London: John Murray; 1859.
